# Pancreatic cystic lesions: position paper of the SIRM-AISP multidisciplinary group

**DOI:** 10.1007/s11547-026-02246-5

**Published:** 2026-06-24

**Authors:** Mirko D’Onofrio, Beatrice Mascarin, Riccardo De Robertis, Laura Romanini, Alfonso Reginelli, Giuseppe Petralia, Nicolò Cardobi, Silvia Carrara, Luca Barresi, Roberto Salvia, Salvatore Paiella, Stefano Francesco Crinò, Luca Frulloni, Claudio Luchini, Nicoletta Gandolfo, Luca Brunese, Giovanni Morana

**Affiliations:** 1 Radiology, GB Rossi Hospital, AOUI Verona, Department of Diagnostics and Public Health, University of Verona, Verona, Italy; 2 University of Campania ‘Luigi Vanvitelli’, Caserta, Italy; 3 Division of Radiology, IEO European Institute of Oncology IRCCS, Milan, Italy; 4 Department of Oncology and Hemato-Oncology, University of Milan, Milan, Italy; 5 Department of Biomedical Sciences, Humanitas University, Via Rita Levi Montalcini 4 20072 Pieve Emanuele, Milan, Italy; 6 IRCCS Humanitas Research Hospital, Via Manzoni 56, 20089 Rozzano, Milan, Italy; 7 Pancreas Unit, Istituto Mediterraneo per i Trapianti e Terapie di Alta Specializzazione - IRCCS ISMETT, Palermo, Italy; 8 University of Pittsburgh Medical Center Italy, UPMC Italy, Palermo, Italy; 9 Pancreatic Surgery Unit, Department of Engineering for Innovation Medicine (DIMI), University of Verona, 37134 Verona, Italy; 10 Pancreatic Surgery Unit, Department of Surgery, Dentistry, Paediatrics and Gynecology, University of Verona, 37134 Verona, Italy; 11 Department of Medicine, Pancreas Institute, University of Verona, Verona, Italy; 12 Diagnostic Imaging Department, IRCCS AOM - Radiology Villa Scassi Hospital, Corso Scassi 1, Genoa, Italy; 13 University of Molise, Campobasso, Italy; 14 Department of Radiology, Teaching Hospital Ca’ Foncello, Treviso, Italy; 15 Direttore SC Radiologia , Direttore Dipartimento dei Servizi Diagnostici, ASST Cremona, Cremona, Italy; 16 Department of Diagnostics and Public Health, Section of Pathology, University of Verona, Verona, Italy

**Keywords:** Pancreatic cystic lesions, Pancreatic cancer, MRI, Follow up, Surgery

## Abstract

Pancreatic cystic lesions represent an increasingly common clinical finding due to the widespread use of cross sectional imaging, encompassing a heterogeneous spectrum ranging from benign entities to premalignant and malignant neoplasms. Their rising incidence poses significant challenges in diagnosis, risk stratification, and management, requiring a careful balance between avoiding unnecessary interventions and preventing malignant progression. This position paper, developed by an intersocietal multidisciplinary panel of experts in pancreatic disease, aims to provide a comprehensive and evidence-based overview of these lesions. Current classifications, imaging features, and differential diagnosis are discussed, with particular emphasis on the role of magnetic resonance imaging. Key entities including serous cystic neoplasms, mucinous cystic neoplasms, intraductal papillary mucinous neoplasms, and rarer cystic tumors are analyzed in terms of biological behavior and radiological characteristics. Furthermore, the document reviews available international guidelines and proposes a pragmatic approach to clinical management, including indications for surveillance, endoscopic evaluation, and surgical treatment. Special attention is given to risk stratification based on high-risk stigmata and worrisome features, as well as to individualized patient management. In conclusion, this paper provides a shared expert perspective to support standardized and personalized management of pancreatic cystic lesions in clinical practice.

**Supplementary Information:**

The online version contains supplementary material available at https://doi.org/10.1007/s11547-026-02246-5.

## Introduction

Pancreatic cystic lesions encompass a broad spectrum of conditions, ranging from inflammatory entities (such as pseudocysts) to benign lesions and neoplasms with variable malignant potential, including aggressive forms. The incidental detection of pancreatic cysts is increasing; population-based studies indicate that pancreatic cystic lesions are diagnosed in approximately 2–3% of abdominal CT examinations and up to 20% of abdominal MRI studies [1, 2]. Their prevalence increases with age [3]. Many of these lesions are completely asymptomatic.

The growing prevalence and increasing complexity of managing these lesions led Gonda TA et al. [4] to describe this phenomenon as a true “tsunami,” emphasizing how the widespread use of abdominal imaging techniques has markedly increased incidental diagnoses. This trend poses significant challenges in risk stratification, clinical management, and healthcare resource allocation. Furthermore, up to 47% of pancreatic cystic lesions have malignant potential [5], which justifies the clinical and radiological interest in accurate identification, risk stratification, and appropriate management.

## Classification

**Fig. 1:**
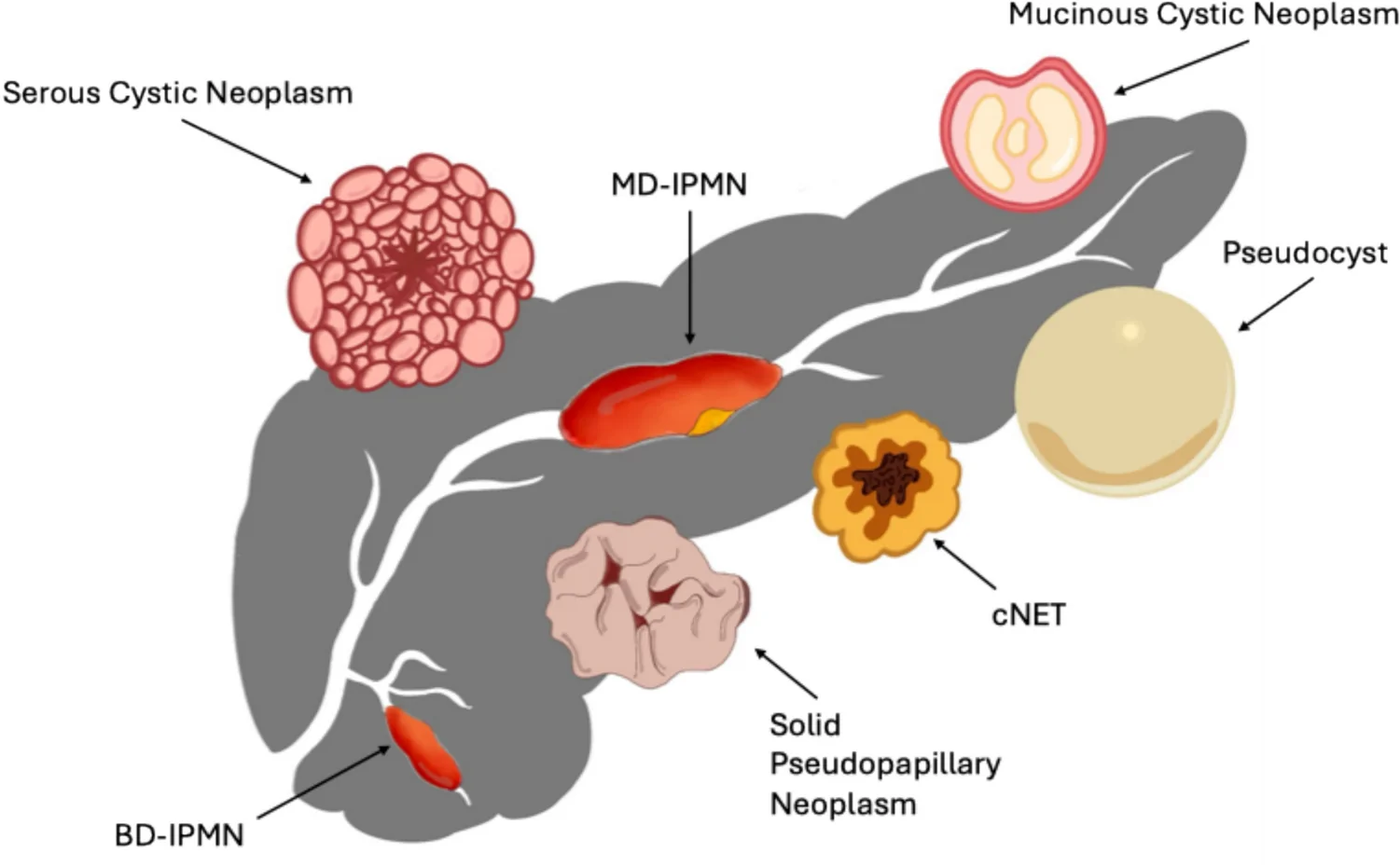
Schematic representation of pancreatic cystic lesions. MD-IPMN = main duct IPMN; BD-IPMN = branch duct IPMN; cNET = cystic neuroendocrine tumor

The sixth edition of the WHO Classification of Digestive System Tumors (2025) [6], updating the previous 2019 edition (Table 1), divides pancreatic cystic lesions into two main categories: neoplastic and non-neoplastic (Table 2) [7]. Each category is further subdivided into epithelial or non-epithelial lesions according to cyst lining and/or histogenesis, resulting in four subgroups: epithelial neoplastic lesions, non-epithelial neoplastic lesions, epithelial non-neoplastic lesions, and non-epithelial non-neoplastic lesions. In clinical practice, the most frequently encountered entities belong to epithelial neoplastic lesions and non-epithelial non-neoplastic lesions (Fig. 1).

**Table 1:** From: WHO classification of digestive system tumors 2019 (5th edition), pancreatic cystic lesions (modified) [6]

Epithelial neoplasms	Non-neoplastic mucinous cyst
Intraductal papillary mucinous neoplasm (IPMN)	Non-neoplastic epithelial lesions
Mucinous cystic neoplasm (MCN)	Lymphoepithelial cyst
Serous cystic neoplasm (SCN)	Enterogenous cyst
Serous cystadenoma associated with von Hippel–Lindau disease	Paraduodenal para-ampullary cyst
Serous cystadenocarcinoma	Ductal retention cyst
Cystic neuroendocrine neoplasm G1-G2	Endometriotic cyst
Acinar cell cystadenoma	Congenital cyst (in malformative syndromes)
Cystic acinar cell carcinoma	Epidermoid cyst of an intrapancreatic accessory spleen
Solid pseudopapillary neoplasm (SPEN, *Frantz tumor*)	Cystic hamartoma
Pancreatic ductal adenocarcinoma, cystic variant	Cystic teratoma (dermoid cyst)
Cystic pancreatoblastoma	
Cystic epithelial metastases	

Non-epithelial neoplasms	Non-neoplastic non-epithelial lesions
Benign non-epithelial neoplasms (e.g., lymphangioma)	Pancreatic pseudocyst
Malignant non-epithelial neoplasms (e.g., sarcomas)	Parasitic cyst

**Table 2:** From: WHO classification of digestive system tumors 2025 (6th edition), pancreatic cystic lesions (modified)

Epithelial neoplasms	Non-neoplastic mucinous cyst
Intraductal papillary mucinous neoplasm (IPMN) and other rarer intraductal lesions, such as intraductal oncocytic neoplasm (IOPN) and intraductal tubulopapillary neoplasm (ITPN)	Lymphoepithelial cyst
Mucinous cystic neoplasm (MCN)	Ciliated foregut cysts
Simple mucinous cyst (SMC)	Paraduodenal para-ampullary cyst
Serous cystic neoplasm (SCN)	Ductal retention cyst
Cystic neuroendocrine neoplasm G1-G2	Endometriotic cyst
Acinar cystic transformation	Congenital cyst (in malformative syndromes)
Cystic acinar cell carcinoma	Epidermoid cyst of an intrapancreatic accessory spleen
Solid pseudopapillary neoplasm (SPN, *Frantz tumor*)	Cystic hamartoma
Cystic epithelial metastases	Cystic teratoma (dermoid cyst)

Non-epithelial neoplasms	Non-neoplastic non-epithelial lesions
Benign non-epithelial neoplasms (e.g., lymphangioma)	Pancreatic pseudocyst
Malignant non-epithelial neoplasms (e.g., sarcomas)	Parasitic cyst

### Pseudocysts

Among non-neoplastic non-epithelial lesions are pancreatic pseudocysts. These are fluid collections containing pancreatic amylase and are typically associated with a clinical history of severe pancreatitis, developing at least 3–4 weeks after symptom onset, which is the time required for a fibrous wall to form around the collection [8]. In other words, the presence of a cystic lesion in a patient with acute pancreatitis should raise suspicion for an underlying cystic neoplasm (potentially the cause of pancreatitis) and should not be misclassified as a pseudocyst.

Pseudocysts are characterized by a well-defined wall and absence of solid or necrotic components [9]. They must be differentiated from IPMN and MCN, as pseudocysts lack epithelial lining and have no malignant potential. On CT or MRI, pseudocysts appear as homogeneous fluid-filled cystic lesions without solid components or debris [10].

Walled-off necrosis (WON) is a late complication of necrotizing hemorrhagic pancreatitis, usually occurring after at least 4 weeks. It presents as a well-encapsulated collection containing both fluid and solid necrotic debris, with a heterogeneous appearance on imaging. It is often multiloculated and, if infected, may show air-fluid levels [9, 11].

Distinguishing between these two entities is crucial for appropriate management: WON may require a surgical approach, whereas pseudocysts, in most cases, do not require invasive treatment except in selected situations. In symptomatic cases, first-line therapy consists of endoscopic drainage, which provides efficacy and safety comparable to surgery, with the advantages of being less invasive and more cost-effective. Surgical intervention is reserved for cases in which endoscopic drainage fails or is technically unfeasible [12].

### Serous cystic neoplasm (SCN)

Serous cystadenoma (SCN) is an epithelial neoplasm with serous content and an indolent biological behavior. It occurs more frequently in females in late adulthood (> 60 years). SCNs typically exhibit slow growth, with a risk of malignant transformation of less than 1% [2, 13].

**Fig. 2:**
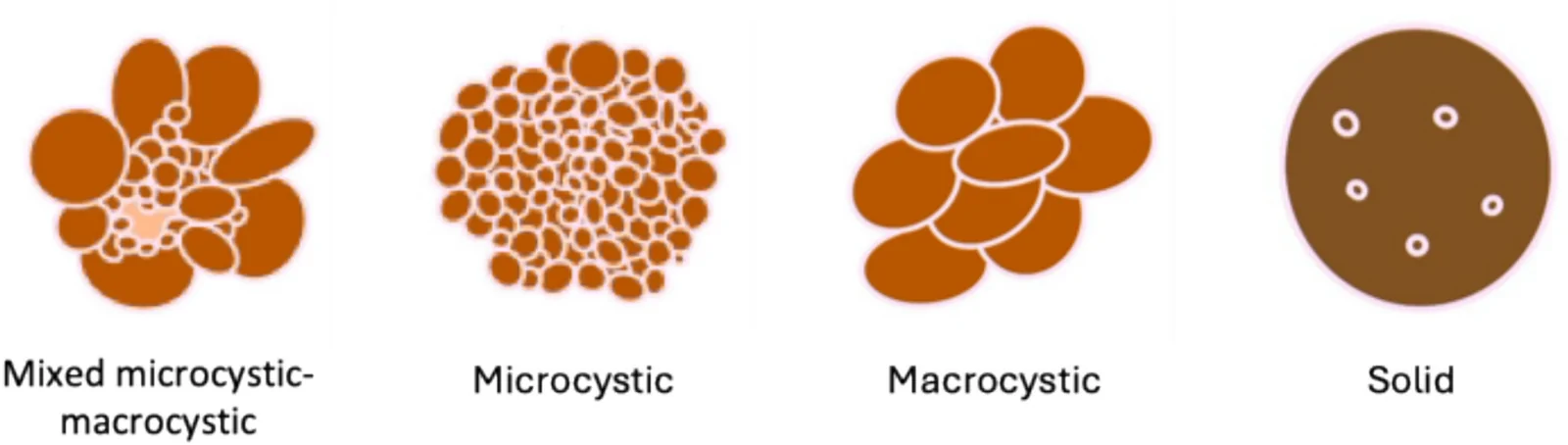
Classification of macroscopic patterns of serous cystadenoma

Four macroscopic patterns are recognized: microcystic, macrocystic, mixed microcystic–macrocystic, and solid (Fig. 2). The microcystic pattern is characterized by multiple cysts smaller than 2 cm separated by thin fibrous septa, producing a honeycomb appearance, often associated with a central fibrous scar and occasional calcifications (sensitivity 32%, specificity 100%) [14]. The macrocystic variant consists of cysts measuring 2 cm or larger, whereas the mixed type combines features of both patterns. The solid variant is characterized by poorly distinguishable microcysts on conventional imaging (e.g., CT), supported by a highly vascularized stroma: on imaging it appears as a hypervascular mass on contrast-enhanced ultrasound (CEUS) and CT, but demonstrates a microcystic architecture on T2-weighted MRI sequences [14].

SCNs may arise in any portion of the pancreas. MRCP sequences typically demonstrate absence of communication with the main pancreatic duct. However, in some cases, the proximity between the SCN and the main pancreatic duct does not allow definitive exclusion of ductal communication, and diagnosis relies mainly on morphological features. When the lesion lies adjacent to the main pancreatic duct, it may exert a compressive effect, potentially leading to upstream obstructive chronic pancreatitis.

### Mucinous cystic neoplasm (MCN)

Mucinous cystic neoplasm (MCN) occurs almost exclusively in middle-aged women (female-to-male ratio ≈ 20:1). It is typically a uni- or oligolocular lesion located in the pancreatic body-tail and lacks communication with the ductal system. MCN contains dense mucinous material and is characterized by thick walls (> 2 mm) lined by mucin-secreting epithelium, with possible septations and peripheral calcifications [15].

On ultrasound, MCN appears as a cystic lesion with anechoic or mildly echogenic content, thick walls, and internal septa; CT demonstrates a well-defined cyst, sometimes with peripheral “eggshell” calcifications; MRI shows a T2-hyperintense, T1-hypointense cystic lesion with walls and septa and no communication with the main pancreatic duct [4, 16]. Differential diagnosis may be supported by cyst fluid analysis obtained by EUS-FNA: elevated carcinoembryonic antigen (CEA) levels and low intracystic glucose values are suggestive of a mucinous lesion [17, 18]. MCN may progress to mucinous cystadenocarcinoma. One imaging feature useful for differentiating SCN from MCN is the peripheral morphology of the lesion: A multiloculated appearance (with or without septa) is typical of SCN, whereas a smooth contour (with or without septa) is more typical of MCN.

A recently described entity, termed simple mucinous cyst, has also been recognized by the WHO classification as a precursor of pancreatic neoplasia. Histologically, the main distinguishing feature from MCN is the absence of ovarian-type subepithelial stroma [19, 20]. Simple mucinous cysts may progress to carcinoma and, compared with MCN, more frequently involve the pancreatic head. These lesions are usually solitary cysts without communication with the main pancreatic duct. They should be considered in the differential diagnosis when a pancreatic cyst larger than 1 cm compatible with a mucinous cystic neoplasm is detected in male patients.

### Intraductal papillary mucinous neoplasm (IPMN)

IPMN is a mucin-producing neoplasm arising from the pancreatic ductal system. The mean age at diagnosis is approximately 60 years [21], and it represents the most common form of pancreatic cystic neoplasm. IPMN is characterized by mucin production by neoplastic epithelium, resulting in ductal dilatation.

Three anatomical subtypes of IPMN are recognized: lesions involving the main pancreatic duct (MD-IPMN, Main Duct type), lesions involving secondary branch ducts (BD-IPMN, Branch Duct type), and mixed-type IPMN, in which dilatation of both the main duct and branch ducts coexist [22]. IPMNs are often multifocal, particularly the BD-IPMN subtype. On ultrasound, IPMN appears as an anechoic or hypoechoic cystic lesion, sometimes with septa or mural nodules, although sensitivity is limited. CT typically demonstrates thin-walled cysts, ductal dilatation, and possible mural nodules or calcifications. MRI/MRCP is the imaging modality of choice for evaluating ductal communication, septations, mural nodules, and main pancreatic duct dilatation, showing high sensitivity in detecting worrisome features (WF) and high-risk stigmata (HRS) [23]. HRS and WF are radiological, clinical, and laboratory criteria identified by guidelines to stratify IPMNs into increasing risk categories (Table 3), guiding patient management [22]. Endoscopic ultrasound (EUS) is the most sensitive technique for detecting mural nodules, with a sensitivity of 83% compared with 53% for CT, increasing to 95% for nodules ≥ 5 mm; specificity is also high, particularly with contrast-enhanced EUS (CE-EUS), reaching values up to 90% [24, 25].

**Table 3:** High-risk stigmata and worrisome features [19]

**High-risk stigmata** (absolute indications; if > 1 present, surgery is indicated in fit patients)
• Obstructive jaundice secondary to the cyst
• Presence of an intracystic nodule or solid component with contrast enhancement ≥ 5 mm
• Main pancreatic duct diameter ≥ 10 mm
• Positive cytology for carcinoma or high-grade dysplasia on cyst fluid analysis (obtained by FNA)
**Worrisome features** (relative indications requiring further diagnostic work-up and possible surgery)
• Cyst ≥ 3 cm
• Mural nodule < 5 mm
• Thickened cyst wall or post-contrast enhancement
• Main pancreatic duct diameter 5–9 mm
• Abrupt change in caliber of the main pancreatic duct with distal parenchymal atrophy
• Suspicious lymphadenopathy
• Elevated CA 19-9
• Cyst growth ≥ 2.5 mm/year
• Acute pancreatitis secondary to IPMN
• New-onset diabetes mellitus within the last yea

In recent years, the concept of a *field defect* has emerged, suggesting that patients with IPMN, particularly multifocal disease, have an increased risk of developing concomitant pancreatic ductal adenocarcinoma (PDAC) in a different pancreatic location from the known cyst, due to a diffuse predisposition of the gland to tumorigenesis [26–28]. Therefore, this risk adds to the intrinsic risk of progression of the cystic lesion itself [18].

These considerations may also apply to rarer intraductal lesions such as intraductal oncocytic papillary neoplasm (IOPN) and intraductal tubulopapillary neoplasm (ITPN). In a non-negligible proportion of cases (up to 30% and 60%, respectively), these lesions may progress to invasive carcinoma [29, 30]. The main differences from IPMN concern the epithelial lining type and the fact that IOPN and ITPN are usually more solid or solid-cystic than classic IPMN.

### Rare cystic neoplasms

Other rare cystic neoplasms include solid pseudopapillary neoplasm (SPEN) and cystic pancreatic neuroendocrine tumor (cPNET).

SPEN is a low-grade malignant cystic neoplasm that almost exclusively affects young women (age < 30 years, typically in the second or third decade) [30]. It accounts for approximately 1–2% of pancreatic neoplasms and is characterized by a combination of solid and cystic components. It typically presents as a large, well-encapsulated mass, with peripheral neoplastic tissue showing a pseudopapillary appearance and an internal cystic architecture resulting from degenerative changes with intralesional hemorrhage. The key diagnostic marker is β-catenin, which demonstrates characteristic nuclear positivity on immunohistochemistry [29].

cPNETs are predominantly non-functioning and therefore often asymptomatic. Radiologically, they appear as uni- or multilocular lesions with a thickened hypervascular wall during the arterial phase, containing fluid due to cystic degeneration. In these cases, Ga-68 DOTATOC PET-CT and EUS-FNA play a crucial role in diagnosis; histological examination allows demonstration of positive neuroendocrine markers such as synaptophysin and chromogranin A [31]. Compared with solid forms, cystic PNETs generally exhibit less aggressive behavior, with lower Ki-67 index, reduced metastatic risk, and a more favorable prognosis.

Rarely (approximately 1–8% of cases), pancreatic ductal adenocarcinoma may present with cystic features [18, 32].

### Differential diagnosis

The differential diagnosis among the various pancreatic cystic lesions should be based on imaging features—including cyst morphology and contour, uni- versus multilocularity, internal content, presence of septa or mural nodules, wall thickness, solitary versus multiple lesions, calcifications, communication with the ductal system, diffusion restriction, and enhancement patterns—in conjunction with clinical data (age, sex, and history of pancreatitis) and laboratory findings (amylase and lipase levels, CEA, and CA 19-9).

MRI represents the imaging modality of choice for the evaluation of pancreatic cystic lesions.

### Patient preparation and MRI study protocols

For patients undergoing MRI evaluation for suspected pancreatic cystic lesions, fasting for at least 4 h is recommended in order to reduce motion artifacts from peristalsis and to optimize distension of the biliary and pancreatic ducts. Oral administration of pineapple or blueberry juice as a negative contrast agent is supported by the literature for suppression of gastric and duodenal signal on MRCP sequences, thereby improving visualization of the biliary and pancreatic ducts. The optimal dose of pineapple juice is approximately 150 mL, to be taken 10–20 min before the examination, while concentrated blueberry juice may represent an alternative provided that it contains an adequate manganese content [8, 33].

The MRI protocol for evaluating pancreatic cystic lesions includes high-resolution multiplanar T2-weighted sequences acquired in at least two orthogonal planes (axial and coronal), pre-contrast fat-suppressed T1-weighted sequences to identify possible solid nodules or hemorrhagic or proteinaceous components, and dynamic contrast-enhanced T1-weighted sequences after gadolinium administration [2]. Three-dimensional and two-dimensional MRCP sequences are indicated for assessing the ductal system and any communication between the ducts and the cystic lesion. Acquisition of MRCP images after contrast administration, in the delayed phase (at least 3 min after injection), is suggested to exploit the T2* effect of gadolinium distributed within the pancreatic interstitium, which further suppresses parenchymal signal and enhances ductal conspicuity, as the ducts do not contain gadolinium.

Diffusion-weighted imaging (DWI) sequences are mandatory and represent an integral part of the pancreatic MRI protocol [34]. The use of echo-planar imaging diffusion-weighted imaging (EPI-DWI), particularly single-shot variants, is recommended due to their rapid acquisition time and ability to minimize respiratory and peristaltic motion artifacts [35].

The use of parallel imaging and simultaneous multi-slice techniques reduces acquisition time, further improving image quality and decreasing artifacts and distortions [36]. Reduced field-of-view (rFOV) EPI sequences, including “zoomed” variants, are particularly useful for evaluating small lesions by increasing spatial resolution.

The inclusion of Dixon sequences is especially helpful in pre- and post-contrast T1-weighted acquisitions, where effective fat suppression improves visualization of cyst walls, septa, mural nodules, and solid components, facilitating enhancement assessment and lesion characterization [37, 38]. Compared with other fat-suppression techniques, Dixon sequences are less sensitive to magnetic field inhomogeneity, often with shorter acquisition times and greater reproducibility.

Hepatobiliary-specific contrast agents are not indicated, as they do not provide additional advantages compared with standard extracellular contrast agents for the evaluation of pancreatic cysts [34, 39]. However, when cystic malignancy is suspected, the use of hepatobiliary-specific contrast agents with delayed biliary excretory phases may improve hepatic staging.

### Advanced molecular profiling and next-generation sequencing of pancreatic cyst fluid

Although the latest ACG guidelines recommend the evaluation of molecular markers on pancreatic cyst fluid only in doubtful cases, their use is crucial in differentiating mucinous cysts [41]. In particular, NGS analysis of intracystic fluid increases sensitivity up to 94% and specificity to 100% [41, 42]. However, these analyses must be performed at third-level centers with technical expertise and multidisciplinary interpretation of the results [43].

Among the analyzed genes is KRAS (Kirsten Rat Sarcoma viral oncogene). Its mutation has a specificity greater than 95% for mucinous cysts [44]. In particular, KRAS mutations have been found in nearly 70% of IPMNs and in 78% of IPMNs associated with invasive adenocarcinoma [44]. At the same time, such mutations are not recognizable in SCNs or cPNETs.

GNAS (Guanine Nucleotide-binding protein, Alpha Stimulating) mutations are useful for the differential diagnosis between IPMN and MCN, as they are found in 39–62% of IPMNs, especially in the intestinal subtype, but are characteristically absent in MCNs [45, 46].

The molecular analysis of KRAS/GNAS mutations performed with NGS has a sensitivity of 89% and a specificity of 100% for mucinous cysts, compared to a sensitivity of less than 65% produced with Sanger sequencing [43]. Furthermore, NGS allows the detection of high-risk mutations in the TP53, SMAD4, CTNNB1 genes and the mTOR pathway, which are strongly associated with advanced neoplasia, including high-grade dysplasia or invasive carcinoma [43]. In this regard, Kannan et al. [47] have demonstrated that these high-risk mutations detected with NGS have a 100% positive predictive value in predicting advanced neoplasia in mixed-type IPMNs, with greater specificity compared to WFs alone.

The PancreaSeq Genomic Classifier represents the most advanced molecular test on cyst fluid. It is based on an NGS platform that evaluates DNA and RNA alterations of 74 genes, including KRAS, GNAS, VHL, MEN1, CTNNB1, and CEA [48]. With this technique, the sensitivity and specificity for mucinous cysts reach 94.6% and 96.4%, respectively, and 86.6% and 97.9% for advanced neoplasia [49]. Moreover, this panel shows high accuracy (sensitivity 97.1–100%, specificity 100%) for rare entities such as IOPN, ITPN, and cPNET [49].

## Clinical framework and management

The increasing number of incidentally detected pancreatic cysts makes it essential to determine which lesions require surveillance and which should undergo surgical treatment. Indeed, many cysts remain stable for years; therefore, from both a cost–benefit perspective and in terms of patient quality of life, unnecessary interventions should be avoided. On the other hand, some lesions may progress to carcinoma, making a proactive strategy appropriate to prevent invasive pancreatic neoplasms, which are known to carry a poor prognosis (5-year survival < 10% in advanced pancreatic cancer) [5].

IPMNs represent a field of intense research: Although often indolent, they may evolve into carcinoma even more than 5 years after the initial diagnosis [50].

Evidence from the literature shows a linear increase in progression risk over time, reaching up to 8% in low-risk cases and up to 25% in intermediate-high-risk IPMNs at 10 years [51, 52]. Moreover, patients with BD-IPMN also demonstrate a cumulative incidence of progression to high-grade dysplasia or carcinoma of approximately 3–5% at 5 years and 8–12% at 15 years from diagnosis [53]. Moreover, patients with IPMN have an increased risk of developing concomitant PDAC, arising independently rather than directly from the IPMN [54], with reported frequencies ranging from 4 to 11%. Therefore, the presence of IPMN is considered a marker of increased risk for high-grade pancreatic neoplasia.

As previously noted, untreated MCNs may evolve into invasive carcinoma in up to 10–17% of cases.

In summary, the growing incidence of pancreatic cyst diagnoses highlights the need for shared guidelines and strategies aimed at personalized management, balancing the risk of malignant progression against treatment-related morbidity. In recent years, several scientific societies have published guidelines on this topic, including the International evidence-based Kyoto guidelines (2024) [22], the European evidence-based guidelines on pancreatic cystic neoplasms (2018) [13], the American Gastroenterological Association Institute guidelines (2015) [55], the American College of Gastroenterology guidelines (2018) [39], and the American College of Radiology White Paper (2017) [2].

This position paper aims to provide a reasoned synthesis and a shared standpoint on pancreatic cystic lesions considering current evidence, defining which patients may safely avoid follow-up.

## Positions in the literature

### Benign lesions and SCNs do not require follow-up

SCNs, as well as other cysts without malignant potential, can be managed conservatively without long-term imaging surveillance once a definitive diagnosis has been established [2, 56]. A confident diagnosis is achieved when typical radiological features are present, such as a microcystic appearance and a central scar or central calcification. If imaging findings are not typical (e.g., macrocystic or solid patterns), further evaluation with EUS and cyst fluid analysis (particularly very low CEA levels) or fine needle biopsy (FNB) is required to differentiate SCN from mucinous or malignant lesions. SCNs typically exhibit slow growth and may remain stable for years [14]; moreover, they are often incidental findings in elderly patients. SCN-related mortality is essentially zero (GRADE 2C) [13], and the risk of transformation into serous cystadenocarcinoma is anecdotal [57].

The European evidence-based guidelines (2018) indicate that, in asymptomatic patients, follow-up may be discontinued after an initial 1-year assessment depending on lesion location and patient age. Similarly, ACR and ACG guidelines recommend discontinuing surveillance once benignity has been confirmed [2, 40].

Surveillance of SCNs with typical imaging features may be performed using MRI without contrast administration [2]; prospective and retrospective studies have shown that the addition of gadolinium does not improve the ability to detect significant changes or risk stratification in SCNs [48, 49]. In particular, a study including more than 300 patients with pancreatic cystic lesions followed for a mean period of 45 months demonstrated excellent interobserver agreement with non-contrast sequences and no additional benefit from contrast administration in the serial evaluation of asymptomatic lesions, including SCNs [58].

In cases of uncertain diagnosis, EUS-FNA may be used to measure CEA levels in cyst fluid: very low CEA values (< 5 ng/mL) are suggestive of SCN, whereas elevated levels (> 192 ng/mL) indicate mucinous cysts [40]. Measurement of intracystic glucose (cutoff < 50 mg/dL) further improves differentiation, as glucose is typically low in mucinous cysts and normal or high in serous lesions. If the diagnosis remains indeterminate after EUS-FNA, advanced techniques such as needle-based confocal laser endomicroscopy may be considered, as they demonstrate high specificity for SCN diagnosis [40].

The only clinical scenario that may require surgical resection of an SCN is significant volumetric growth leading to compressive symptoms (e.g., jaundice, pancreatic duct dilatation, or pain).

In conclusion, follow-up of SCNs should be performed using MRI without contrast, as only dimensional changes and relationships with adjacent structures need to be monitored.

### MCNs may be followed over time

Pancreatic MCNs represent an important clinical entity, as surgical series report that approximately 10–17% of resected MCNs already harbor invasive carcinoma or high-grade dysplasia [2]. Given that these lesions are usually solitary, premalignant or potentially malignant, and often detected in relatively young patients, the recommended treatment is primarily surgical, frequently in the form of distal pancreatectomy with splenectomy according to the typical location; at minimum, surgical evaluation is mandatory in all cases. Current guidelines [2, 55] recommend resection of all MCNs ≥ 4 cm in surgically fit patients, particularly when mural nodules, enhancing walls, or elevated CA 19-9 levels are present. Recurrence after complete resection is rare [56]. Postoperative follow-up after curative surgery may be omitted when the diagnosis of mucinous cystadenoma is confirmed.

Surveillance may be proposed for MCNs < 4 cm without mural nodules, without enhancing walls, without CA 19-9 elevation, and showing stability over time, as this subgroup is associated with minimal malignancy risk due to a low probability of high-grade dysplasia or invasive carcinoma [57, 58].

Patient preferences should clearly be considered. Surveillance with contrast-enhanced MRI or EUS depends on lesion size: every 6–12 months for MCNs ≥ 2 cm and every 1–2 years for smaller, stable lesions [59]. After resection of an MCN, in the absence of procedures associated with risk of tumor seeding and when the lesion has been completely removed intact (especially when only adenoma is present), surveillance of the remaining pancreatic parenchyma is not required given the typically unifocal nature of the disease.

### Follow-up of IPMNs is indicated in surgically fit patients

IPMNs carry a risk of progression to invasive carcinoma even many years after diagnosis. For low-risk BD-IPMNs, the cumulative probability of developing pancreatic carcinoma is approximately 3% at 5 years, 8% at 10 years, and up to 15% at 15 years from diagnosis, with a risk that increases linearly over time and persists beyond the first five years of follow-up [50, 60, 61]. In IPMNs that are not low risk due to the presence of HRS or WF, the risk of malignant transformation is significantly higher, reaching nearly 25% at 10 years. MD-IPMNs show malignancy rates ranging from 33 to 85% in surgical series [4]. Furthermore, the literature demonstrates that malignancy risk increases progressively with the number of WF: approximately 22% with a single WF, rising to 59% with three and reaching even higher values when four or more WF are present [62].

When IPMN is suspected, diagnosis is largely presumptive and based on MRI identification of cystic lesions that appear to communicate, more or less clearly, with the ductal system. Therefore, histotype percentages reported in the literature should be applied cautiously to radiological practice, where small or clinically insignificant cystic lesions not truly representing IPMN are not uncommon [63].

Possible management strategies for IPMN include surgical resection, active surveillance, or discontinuation of follow-up. Decision-making should consider several factors, including estimated malignancy risk, overall patient condition (performance status, age, surgical eligibility, and comorbidities), family history of pancreatic neoplasia, and germline mutations.

Most guidelines recommend that patients with IPMN and HRS who are eligible for surgery undergo resection [4]. For intermediate-risk IPMNs, EUS, with or without FNA, improves risk stratification by guiding the decision between surgery and surveillance and by providing material for cytological and molecular analysis [22, 64]. EUS is recommended in IPMNs with WF to more accurately identify mural nodules, wall thickening, and to perform FNA of cyst fluid or solid components, improving sensitivity compared with MRI alone [64].

In surgically fit patients, segmental resection of the involved pancreatic portion is often performed (pancreaticoduodenectomy, distal pancreatectomy, or rarely total pancreatectomy). Two-stage total pancreatectomy may be considered in cases of positive resection margins. Surgical indication is primarily based on the presence of HRS or imaging findings strongly suggestive of carcinoma, regardless of IPMN size [22, 65, 66]. The presence of WF alone does not constitute an absolute indication for surgery, and in the absence of HRS, surveillance with MRI, with or without EUS, remains the preferred strategy.

After resection of an IPMN, surveillance of the remaining pancreatic parenchyma is required, even in the absence of high-grade dysplasia on histology, due to the multifocal nature of the disease [22].

All guidelines agree that follow-up is not indicated in patients who are not candidates for surgery, as surveillance would not provide therapeutic benefit and would represent an unnecessary use of healthcare resources.

In other low-risk IPMN cases, follow-up typically includes an initial control at 6 months after diagnosis, followed by annual or biennial imaging, or intervals tailored to cyst size (using the largest lesion in multifocal disease) according to different guidelines. The interval may be extended or even discontinued if the lesion—particularly when < 2 cm—remains stable and without WF for at least 5 years [22].

Regarding duration of follow-up, current evidence does not unequivocally support discontinuation of surveillance, as progression may occur even after the first 5 years [67]. The AGA guidelines and recent Kyoto guidelines suggest that surveillance discontinuation may be considered in patients with BD-IPMN < 20 mm in diameter, without HRS or WF, and stable for at least 5 years [22, 55]. Conversely, discontinuation is not recommended in young patients or those with familial or genetic predisposition to pancreatic neoplasia. The Kyoto guidelines emphasize that the decision to stop surveillance should be individualized and shared with the patient [22].

It is also well established that patients with IPMN have a 3–fivefold increased risk of developing PDAC compared with the general population [60]. The Dual Carcinogenesis Theory proposes that PDAC may arise either from areas of high-grade dysplasia within the IPMN or independently elsewhere in the pancreas, facilitated by genomic instability and a pro-inflammatory microenvironment affecting the entire gland [50, 68, 69].

Currently, in patients who are potential surgical candidates with a life expectancy greater than 10 years, maintaining periodic MRI surveillance is considered the most prudent approach [67]. At the same time, cost-containment strategies should be adopted, such as early surgical evaluation to avoid follow-up in patients who are not candidates for curative intervention and the use of abbreviated MRI protocols. Recent literature supports short MRI protocols for IPMN surveillance, particularly when interpreted by radiologists specialized in pancreatic disease. These protocols should include at least T2-weighted, T1-weighted, DWI, and MRCP sequences, with dynamic post-contrast phases reserved for cases of suspected progression [70]. Several studies have demonstrated that such protocols allow accurate assessment of IPMN size, main pancreatic duct caliber, mural nodules, and WF/HRS, with diagnostic performance comparable to standard protocols but with significant reductions in time and cost [70, 71].

### Management of rare forms (cPNET, SPEN)

Once diagnosed, SPEN should always be referred for surgical evaluation, regardless of size or location, due to its potential for distant metastasis and the excellent prognosis following R0 resection. Surgical approaches should prioritize parenchyma-sparing techniques, particularly in young patients and in the absence of vascular invasion [31]. Prognosis after resection is excellent, with 5-year survival rates exceeding 95% and a low risk of recurrence [31].

For cystic pancreatic neuroendocrine tumors (cPNETs), surgical decision-making mainly depends on lesion size and tumor grading (Ki-67 index). The National Comprehensive Cancer Network recommends that in patients with small (≤ 2 cm), well-differentiated (grade 1) cPNETs, active surveillance with cross sectional imaging (CT or MRI, with optional Ga-68 DOTATOC PET-CT) every 6–12 months during the first 1–2 years is appropriate, followed by annual imaging if the lesion remains stable [72]. cPNETs larger than 2 cm are generally resected to prevent metastatic spread. For metastatic or unresectable disease, medical therapies such as somatostatin analogs, targeted therapies, and peptide receptor radionuclide therapy (PRRT) are available.

After complete resection of a cystic cPNET with Ki-67 > 5% or positive lymph nodes, surveillance consists of MRI every 6–12 months for 3 years, followed by imaging every 1–2 years up to 10 years [73].

**Fig. 3:**
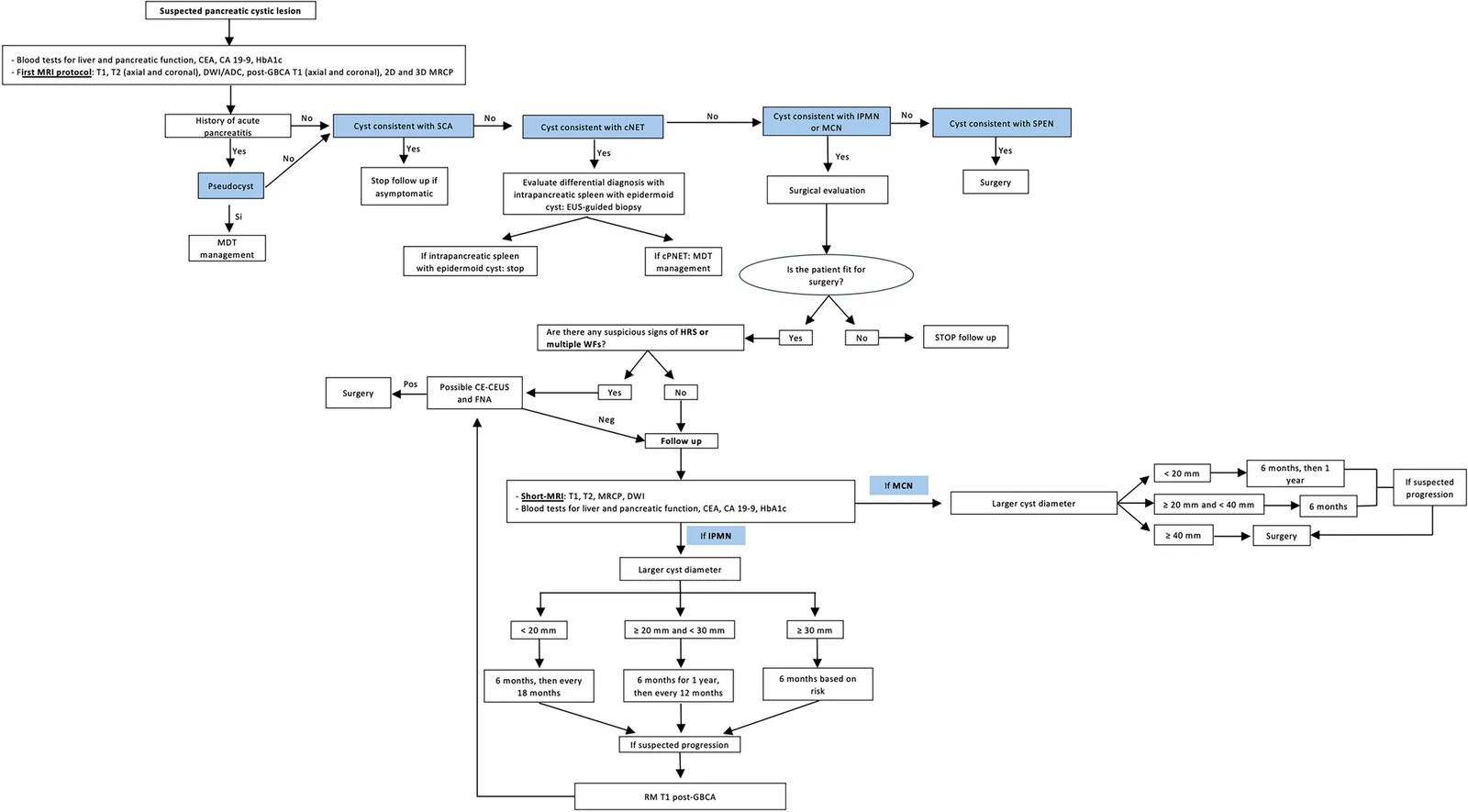
Management algorithm for pancreatic cystic lesions

A management algorithm is proposed in Fig. 3.

### Structured MRI reporting template for pancreatic cystic lesions

The complexity in the management of pancreatic cystic lesions requires clear and unambiguous radiological communication. The introduction of a structured report based on standardized morphological and clinical parameters allows to overcome the limitations of traditional descriptive reporting, effectively supporting clinical and surgical decision-making. An intersocietal proposal for a structured report for MRI is provided in the supplementary materials.

## Conclusions

Pancreatic cystic lesions represent an increasingly frequent diagnostic and management challenge in clinical and radiological practice. Key recommendations for the management of these conditions include careful multidisciplinary evaluation to ensure appropriate, efficient, and dedicated diagnostic and therapeutic care pathways.

## Supplementary Information

Below is the link to the electronic supplementary material.

Supplementary file1 (DOCX 20 kb)

## Abbreviations

ACG: American college of gastroenterology
ACR: American college of radiology
AGA: American gastroenterological association
AISP: Associazione Italiana per lo Studio del pancreas
BD-IPMN: Branch duct IPMN
CA 19-9: Carbohydrate antigen 19-9
CE-EUS: Contrast-enhanced endoscopic ultrasound
CEA: Carcinoembryonic antigen
CEUS: Contrast-enhanced ultrasound
cPNET/cNET: Cystic pancreatic neuroendocrine tumor
CWRM: MR cholangiopancreatography (negative MRCP)
DWI: Diffusion-weighted imaging
EPI-DWI: Echo-planar imaging diffusion-weighted imaging
EUS-FNA: Endoscopic ultrasound fine needle aspiration
EUS: Endoscopic ultrasound
HRS: High-risk stigmata
IPMN: Intraductal papillary mucinous neoplasm
MCN: Mucinous cystic neoplasm
MD-IPMN: Main duct IPMN
MRCP: Magnetic resonance cholangiopancreatography
PDAC: Pancreatic ductal adenocarcinoma
PET-CT: Positron emission tomography-computed tomography
rFOV: Reduced field of view
MRI: Magnetic resonance imaging
SCN: Serous cystic neoplasm
SIRM: Società Italiana di radiologia medica ed interventistica
SPEN: Solid pseudopapillary neoplasm
CT: Computed tomography
WF: Worrisome features
WHO: World Health Organization
WON: Walled-off necrosis
