# Supplementary material for: Pancreatic cystic lesions: position paper of the SIRM-AISP multidisciplinary group
Source: Radiol Med. 2026 Jun 24;131(9):1439–51. doi: 10.1007/s11547-026-02246-5 (PMC13658329; doi:10.1007/s11547-026-02246-5)
Supplement: Supplementary file 1 [file 11547_2026_2246_MOESM1_ESM.docx]

# Structured MRI reporting template for pancreatic cystic lesions

**CLINICAL INFORMATION**

- Indication: Incidental finding / Surveillance / Symptom evaluation
- Relevant history: Pancreatitis / Jaundice / Weight loss / Diabetes particularly of new onset / Family history of pancreatic cancer / Prior pancreatic surgery
- Prior imaging: Date and modality of comparison study
- Relevant labs or FNA / FNB if available: CA 19-9, HbA1c, liver and pancreatic function test.

**TECHNIQUE**

- MRI field strength: 1.5T / 3T
- Sequences performed: T1W in and out-phase pre-contrast (and T1W post-contrast), T2W, T2 FS, DWI, MRCP (2D and/or 3D)
- Contrast agent: Agent, Dose
- Limitations: Motion artifact / Incomplete sequences / Other

**FINDINGS**

**1. Cyst(s) identification and morphology**

- Number of cysts: Single / Multiple

- Morphology: Unilocular / Oligolocular / Multilocular (microcystic vs. macrocystic) / Mixed solid-cystic

- Internal architecture: Simple fluid / Septations (thin 2 mm / thick ≥2 mm) / Debris / Hemorrhagic signal

- T1 signal: Hypointense / Hyperintense (suggestive of mucin or hemorrhage)

- T2 signal: Hyperintense / Heterogeneous

- Diffusion restriction: Present / Absent / Indeterminate

- Wall characteristics: Thin / Thickened / Enhancing (specify thickness in mm)

- Calcifications (if visible): peripheral (eggshell) / central / mural / none identified on MRI.

**2. Cyst(s) anatomical location**

- Location: Head / Uncinate process / Isthmus / Body / Tail
- Communication with MPD: definite / suspected / not identified

**3. Cyst(s) size and eventual interval change**

Growth threshold flag: ≥20% increase or ≥2.5 mm/year → WF

**4. Main pancreatic duct assessment**

- MPD diameter: _____ mm (measure at head, body, tail)
  - Head: mm | Body: mm | Tail: ___ mm
- MPD morphology: Normal / Diffusely dilated / Segmentally dilated / Abrupt caliber change
- Distal pancreatic atrophy: Present / Absent

**5. Mural nodules and solid components**

- Mural nodule: Present / Absent - If present: Size ___ mm | Enhancing: Yes / No
- Solid component within cyst: Present / Absent - If present: Size ___ mm | Enhancement pattern: Avid / Mild / None

**6. Risk stratification: high risk stigmata or worrisome features** (check all that apply)

High risk stigmata:

- Enhancing solid component ≥5 mm within cyst
- MPD diameter ≥10 mm
- Obstructive jaundice with cyst in pancreatic head
  - Evaluate biliary dilation: Present (CBD diameter ___ mm) / Absent

Worrisome features:

- Acute pancreatitis secondary to the cyst
- Cyst diameter ≥30 mm
- Thickened or enhancing cyst wall
- Mural nodule < 5mm
- MPD diameter 5-9 mm
- Lymphadenopathy: Present (location, short axis) / Absent
- Abrupt MPD caliber change with distal atrophy
- Pancreatic parenchyma: Normal / Atrophic / Fatty replacement / Chronic pancreatitis changes

Other:

- Vascular involvement: SMA / SMV / portal vein / celiac trunk / splenic vassels - specify contact, encasement, or occlusion and eventual thrombosis
- Liver lesions
- Other relevant findings

**IMPRESSION**

1. Cyst characterization: favored diagnosis (BD-IPMN / MD-IPMN / Mixed-type IPMN / MCN / SCA / cPNET / Pseudocyst / SPN / Other )

- Confidence level: High / Moderate / Low
- Differential diagnosis: List if applicable

2. Risk category: none worrisome features or high-risk stigmata / worrisome feature(s) present / high-risk stigmata present

3. Interval change (if comparison available): Stable / Increased size / New worrisome features / New high-risk stigmata

4. Recommendation:

- Continued surveillance with MRI/MRCP in ___ months (specify interval)
- EUS with FNA recommended
- Multidisciplinary / surgical consultation recommended
- No further follow-up recommended (specify reason)
- Other: _______________
